# Central nervous system acting medication used by young adults with borderline personality disorder in Sweden 2006–2019

**DOI:** 10.1177/02698811251413479

**Published:** 2026-02-16

**Authors:** Annika Tiger, Yasmina Molero, Isabell Brikell, Zheng Chang, Paul Lichtenstein, Henrik Larsson, Brian M D’Onofrio, Clara Hellner, Tyra Lagerberg, Nitya Jayaram-Lindström, Ralf Kuja-Halkola

**Affiliations:** 1 Centre for Psychiatry Research, Department of Clinical Neuroscience, Karolinska Institutet & Stockholm Health Care Services, Sweden; 2 Department of Medical Epidemiology and Biostatistics, Karolinska Institutet, Stockholm, Sweden; 3 Department of Biomedicine, Aarhus University, Denmark; 4 Department of Global Public Health and Primary Care, University of Bergen, Norway; 5 School of Medical Sciences, Örebro University, Sweden; 6 Department of Psychological and Brain Sciences, Indiana University, Bloomington, USA; 7 Department of Psychiatry, Warneford Hospital, University of Oxford, UK

**Keywords:** borderline personality disorder, personality disorders, pharmacology, central nervous system, medication, drugs for anxiety, drugs for insomnia, drugs for depression, drugs for psychosis, drugs for relapse prevention

## Abstract

**Background:**

Most individuals diagnosed with borderline personality disorder (BPD) use psychotropic medications. BPD symptom severity is elevated in young adulthood, but medication utilisation studies in young adults with BPD are lacking.

**Aims:**

To investigate the use of central nervous system (CNS) acting medications among young adults (aged 18–24), who were diagnosed with BPD, focusing on the years around the first established diagnosis.

**Methods:**

A cohort study of all individuals with a registered BPD diagnosis in Sweden was conducted using national population register data. Dispensed prescriptions between July 2005 and June 2020 were utilised. Prevalent use, long-term use, and co-medication were investigated during specific 1-year periods: the year before, and then the first, second, and third year after diagnosis.

**Results:**

Medication use peaked for all three measures in the year following the first BPD diagnosis: 90.3% used CNS-acting medication during that year, and antidepressants were the most used group (70.1%). Furthermore, 74.5% used medication for >12 months, and 34.8% used ⩾3 medication groups in co-medication. Compared to a reference group of individuals diagnosed with depression, the BPD group used more medication. In the depression group, 87.5% used medication, 55.2% used medication for >12 months, and 3.8% used ⩾3 medication groups in co-medication. Moreover, medication use increased from 2006 to 2019.

**Conclusion:**

This indicates the importance of continuously evaluating risks and benefits with medication use, and in having easy access to up-to-date guidance about best clinical practice for individuals diagnosed with BPD, to facilitate appropriate and safe medication use.

## Introduction

Borderline personality disorder (BPD) is a condition characterised by emotional instability, interpersonal difficulties, and impulsive behaviours, often including angry outbursts, substance use, self-harm, and suicidal acts. Personal suffering and societal costs associated with BPD are substantial (Hastrup et al., 2019), with global prevalence estimated to 2% in adolescents and in adults (Winsper et al., 2020). In the Swedish National Patient Register data, cumulative incidence up to age 40 is over 1%, with an overrepresentation among women (almost 2% and 0.4% in men; Skoglund et al., 2019).

Psychotherapy is the recommended first-line treatment for BPD in international guidelines (Ekselius, 2017; Keepers et al., 2024; National Collaborating Centre for Mental Health, 2009; National Health and Medical Research Council, 2012; Simonsen et al., 2019; Storebø et al., 2020). Several types of psychotherapy are effective in reducing symptoms and increasing functioning in individuals with BPD. Dialectic behaviour therapy and mentalisation-based treatment are among the most common and well-researched types (Stoffers-Winterling et al., 2022b).

In the Swedish clinical guidelines (Ekselius, 2017), no specific medication is recommended for the treatment of BPD. However, pharmacotherapy is recommended to be considered adjunctive to psychotherapy for complicating comorbid conditions, such as depression, and in severe crises. The clinical guidelines advise against the use of pharmacological agents with addictive potential, for example, benzodiazepines and opioids, and recommend evaluating any prescribed medications regularly to monitor and discontinue in the absence of beneficial effects. This is in line with international guidelines (Keepers et al., 2024; National Collaborating Centre for Mental Health, 2009; National Health and Medical Research Council, 2012) and is reflective of risks with medication use in this patient group, such as deliberate intoxication (Piotrowska et al., 2019), and of the overall weak evidence for effectiveness of pharmacological treatments in BPD. This applies to effects of medications used to treat the full BPD condition, its sub-symptoms, for example, self-harm, impulsivity, and aggression, commonly associated psychopathology (e.g., depression), as well as to evidence on the adverse effects of medications used by individuals diagnosed with BPD (e.g., risk of weight gain and poisoning; Gartlehner et al., 2021; Stoffers-Winterling et al., 2022a; Stoffers et al., 2010).

Studies from the UK, Spain and the US have shown that most individuals with BPD receive psychopharmacological treatment, often for long durations (e.g., 58% used antidepressants 16 years after established BPD) and with multiple pharmacotherapeutic agents used concomitantly (e.g., 50% used ⩾3 substances) (Pascual et al., 2021; Paton et al., 2015; Zanarini et al., 2015). The Spanish study also found a decline in benzodiazepine use and an increase in the use of second-generation antipsychotics between the periods 2001 and 2020 (Pascual et al., 2021). Previous studies suffer from several limitations, such as small samples (*N* = 2600, 620, and 290 in the UK, Spanish, and US studies, respectively), populations restricted to patients at specific units, comparisons only to individuals with other personality disorders and cross-sectional designs. This reduces how generalisable the findings are and makes it harder for clinicians to comprehend the extent of medication use in the BPD group and how it compares to other groups in specialist psychiatric services. It also limits the understanding of how the use changes over time in relation to the time when BPD is diagnosed. When it comes to manifestations of BPD at different ages, previous studies have shown that symptom severity and difficulties are elevated in early adulthood (Bornovalova et al., 2009; Solmi et al., 2022). However, studies of medication use in young adults diagnosed with BPD are currently lacking.

Given the limitations in previous studies, large-scale, population-representative studies are needed to clarify patterns of medication use in young adults with BPD.

The aim of the present study was to characterise patterns of use of central nervous system (CNS) acting medications, including medications licensed for both psychiatric and non-psychiatric indications, by examining dispensed prescriptions among individuals aged 18–24 who have been diagnosed with BPD in the total Swedish population. The primary aim was to investigate patterns of use around the time of receiving the first BPD diagnosis (the year before, and then the first, second, and third years after diagnosis). Secondary aims were to investigate changes in medication use patterns over calendar years (investigating sample years, i.e., 2006, 2010, 2014, and 2019) among individuals with a prior diagnosis of BPD, and to compare these patterns with those observed in individuals diagnosed with depression—a common, and clinically well-characterised condition (Lundberg et al., 2022) and hence of value as a clinical reference group.

## Methodology

### Data sources

This study was based on data from several Swedish national registers linked with each individual’s unique personal identity number. The National Patient Register (NPR) holds information on diagnoses established in inpatient care since 1973 and outpatient specialist care since 2001 in Sweden (Ludvigsson et al., 2011). We used diagnoses registered according to the International Classification of Diseases, Tenth Revision (ICD-10) from 1997 and later. The Prescribed Drug Register contains information on the dispensation date for all drugs dispensed at pharmacies in Sweden from 1st of July 2005, onwards. Drugs are coded according to the Anatomical Therapeutic Chemical (ATC) classification. The Cause of Death Register includes data on dates and causes of death, and the Total Population Register on migrations (Ludvigsson et al., 2016).

### Study population

The study population consisted of individuals with diagnosed BPD, defined as those with a registered F60.3 ICD-10 code (emotionally unstable personality disorder) in the NPR at age 15–64 and who were young adults (aged 18–24) during the investigated years. We included both primary and secondary diagnoses. The time of the first observed diagnosis was considered the date of being diagnosed. Diagnoses registered in the NPR are formally coded according to the ICD, although the actual clinical diagnosis is defined as in the Diagnostic and Statistical Manual for Mental Disorders (DSM), as customary in Sweden and in the rest of Europe (Kouppis and Ekselius, 2020), wherefore we refer to the condition as BPD. A previous medical chart validation study found a positive predictive value of 100% using ICD-criteria to diagnose BPD, in individuals aged 15–64 (Kouppis and Ekselius, 2020).

Medication use was studied using data from July 1st, 2005, or from age 13 onwards, whichever occurred later, until June 30th, 2020, death, or emigration, whichever occurred first. Individuals who immigrated after 2005 or age 13 were excluded (see Supplementary Figure 1 for cohort selection).

To facilitate interpretation of the results, we defined a reference group consisting of individuals commonly encountered within the health care system and with a similar age at disorder onset, that is, individuals diagnosed with depression in psychiatric specialist care (ICD-10 code F32 (depressive episode) or F33 (major depressive episode, recurrent)), as registered in the NPR. They were randomly selected by matching one individual with depression (without any BPD diagnosis) to one with BPD with regard to sex, year of birth, year of diagnosis and being born in Sweden or not.

### Measures

The use of CNS-acting medication was defined as dispensed prescriptions of systemic-acting CNS medications. Those were substances from the N-chapter of the ATC codes, plus guanfacine licensed for attention deficit/hyperactivity disorder (ADHD). We defined groups of medication based on how the substances are of relevance in psychiatric care, while also taking pharmacological mechanisms of action into account using the ATC classification and terminology endorsed by the World Health Organization. This approach was chosen as the ATC classification system was developed to facilitate comparisons in drug use between geographic regions and as it simplifies comparisons to previous studies that have used this approach (WHO Collaborating Centre for Drug Statistics Methodology, 2024). To further clarify, and to align with the more recent pharmacology-driven neuroscience-based nomenclature (NbN) (Nutt and Blier, 2016), overarching NbN terms were also mentioned when applicable.

For this study, we were interested in eight main groups (and seven subgroups) of medication (see Supplementary Table 1 for specific ATC codes, rationale for groupings, and NbN terms and groupings when applicable) (overarching NbN equivalent terminology in square brackets): 1) Antidepressants [drugs for depression], 2) Antipsychotics [drugs for psychosis], 3) Anxiolytics, hypnotics and sedatives (AHS) [drugs for anxiety and insomnia], 4) ADHD medications, 5) Substance use disorder medications, 6) Opioids, 7) Antiepileptics/mood stabilisers [drugs for relapse prevention] and 8) Antiparkinson medications.

We also investigated all eight groups together as “any CNS-acting medications.”

For analyses of medications of specific interest, seven additional subgroups were defined: 9) Benzodiazepines, 10) Selective serotonin reuptake inhibitors (SSRIs), 11) Serotonin and norepinephrine reuptake inhibitors (SNRIs), 12) Second-generation antipsychotics, 13) First-generation antipsychotics, 14) Lamotrigine [glutamate voltage-gated sodium channel blocker], and 15) Lithium [lithium enzyme modulator].

Three measures for patterns of medication use (prevalence of use, duration, and co-medication) were defined as follows:

Prevalence of use was the proportion of the total group of individuals with BPD who had filled a prescription during each investigated year.

Duration of use was defined as the period during which the prescribed medication was continuously dispensed, provided that the interval between two dispensed prescriptions did not exceed 6 months (Lichtenstein et al., 2012; Zetterqvist et al., 2013). The starting time was the date of a dispensed prescription that followed a gap of at least 6 months. Treatment was considered to end at the time of the last filled prescription plus an additional period equal to the interval between the second-to-last and last filled prescription. Durations were categorised as single prescription, short term (⩽6 months), medium term (6–12 months) or long term (>12 months).

Co-medication was investigated quantitatively and qualitatively. Quantitative co-medication was defined as the highest number of medication groups used concomitantly (out of the eight main groups) for a time lasting 30 days or more. We observed the highest number of medications used during any 30-day period with rolling 10-day intervals. For qualitative co-medication, we then identified the 10 most common combinations (i.e., medications used in monotherapy and in combination for 30 days or more) out of the eight main groups of CNS-acting medications.

Comorbid psychiatric conditions were defined as registered ICD-10 diagnoses during the year before and the year after being diagnosed with BPD and were identified to aid in the interpretation of the results (see Supplementary Table 2 for specific conditions and ICD-10 codes).

### Statistical analyses

Different one-year periods of time were investigated between 2006 and 2019 with regard to the patterns of medication use. All reported uses are for separate sub-cohorts of individuals who were young adults (aged 18–24) during most part of each investigated year (i.e., ⩾17.5 and <24.5 at the start of each investigated year). The total study cohort consisted of those who were young adults during the investigated one-year periods, regardless of the time or age when BPD was first diagnosed. The main study cohort was a subgroup of that group in whom the year prior to being diagnosed with BPD, the first year after the first BPD, the second year after BPD, or the third year after BPD occurred between 2006 and 2019 (Table 1).

**Table 1.** Description of study population of young adults diagnosed with BPD (born 1981–2001).

	Overall	Female	Male
Total study cohort (*n*)	18,426	16,162	2,264
Age at diagnosis			
Mean (sd)	23.7 (4.3)	23.5 (4.3)	24.9 (4.4)
Range	15.0–38.3	15.0–38.3	15.1–38.3
Year diagnosed			
Range	1997–2019	1997–2019	1997–2019
Main study cohort (*n*)*	12,266	10,995	1,271
Age at diagnosis			
Mean (sd)	21.4 (2.2)	21.3 (2.2)	22.0 (2.1)
Range	15.5–25.5	15.5–25.5	15.8–25.5
Year diagnosed			
Range	2004–2019	2004–2019	2004–2019

Total study cohort: young adults investigated during the years around the first BPD diagnosis (i.e., the year before, the first-, second-, and third year after diagnosis) and during sample years 2006, 2010, 2014, and 2019.

Main study cohort: young adults investigated during the years around the first BPD diagnosis (i.e., the year before, the first, second, and third year after diagnosis).

* Sub-cohort of total study cohort.

#### Patterns of use before and after BPD diagnosis

First, patterns of use were evaluated the year before and then 1, 2, and 3 years after BPD diagnosis for individuals in whom those years occurred any time between 2006 and 2019. This time period was chosen to examine the same years for the three measures of pattern of use (measures of duration and co-medication required +/− 6 months for any time period examined). For duration, only individuals with full follow-up time in a specific year were included to not overestimate long durations.

We then analysed patterns of use by comparing proportions of prevalent users, of long-term users (>12 months), and mean degree of quantitative co-medication during the year after the first established BPD diagnosis to the year before diagnosis, and the year after the first established BPD diagnosis to the third year after established diagnosis.

#### Comparison with reference group

Patterns of medication use were also compared with those of the matched group diagnosed with depression. First, the patterns of use were estimated for the year before and then the first, second, and third years after depression diagnosis (the same way as for BPD). We compared BPD with depression by analysing the patterns of use in the year after first being diagnosed.

#### Patterns of use over follow-up years (i.e., sample years 2006, 2010, 2014, and 2019), after BPD diagnosis

We assessed patterns of use for the sample years 2006, 2010, 2014, and 2019. Analyses for calendar year trends of prevalent use, long-term use, and mean degree of co-medication were performed. This was done first in individuals who were young adults in the sample years, regardless of the time of first established diagnosis, and then restricted to the subgroup of young adults who had received a first BPD diagnosis at any time before each of those specific sample years.

When statistically testing differences (comparing the year after first BPD and the year after first depression) and changes (comparing the year after first BPD diagnosis to the year before and to the 3rd year after BPD and changes from 2006 to 2019 in the restricted and non-restricted groups), we used generalised estimating equations with a cluster robust sandwich estimator for standard errors, appropriately accounting for some individuals contributing to several measurements for some analyses. We used the identity link for all analyses. Statistical analyses were carried out in females and males together; descriptive data for BPD were additionally presented with stratification by sex.

We adjusted for multiple testing (165 comparisons) using familywise error rate (FWER) test according to Hochberg, resulting in a *p*-value threshold of <0.001, with 120 comparisons being statistically significantly different.

For data management, analyses, and graphics, we used R. Packages utilised were “tidyverse,” “lubridate,” “summarytools,” “drgee,” “ggplot2” and “ggrepel.”

## Results

### Study population

We identified 18,426 individuals with a BPD diagnosis, who were young adults during the investigated years (2006–2019; Table 1). Among those, 12,266 (89.6% female, ages at first diagnosis ranging between 15.5 and 25.5, and years of first diagnosis ranging between 2004 and 2019) were investigated for use during the years of primary interest, the years around the first BPD diagnosis (i.e., the year before BPD, and the first, second, and third year after BPD).

### Patterns of use during different 1-year periods of time

#### Patterns of use before and after BPD diagnosis

There was a peak in all three measures of use during the year following the first established BPD.

##### Prevalence of use

**Figure 1.**
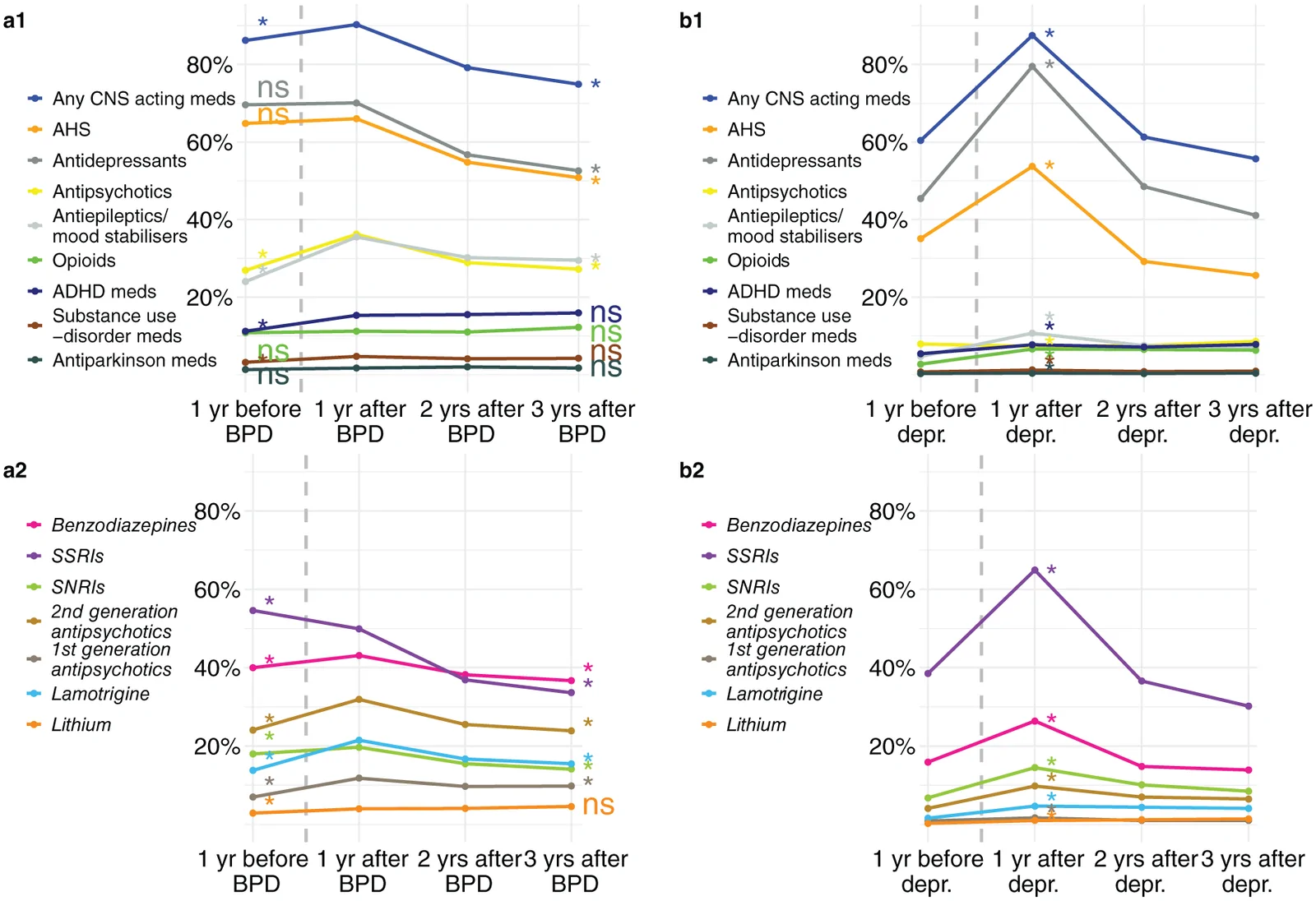
Prevalent medication use in individuals diagnosed with BPD, who were 18–24 years old in the year before, and then 1, 2, and 3 years after first registered BPD diagnosis. Including data on the same respective years and age in matched individuals diagnosed with depression for comparison. *Note*. : (a1/a2): Prevalent use of medication in individuals diagnosed with BPD. Linear regressions, comparing the reference year (year after first established BPD diagnosis) to the year before and the third year after established BPD diagnosis. (b1/b2): Prevalent use of medication in individuals diagnosed with depression. Linear regression comparing the reference year (the year after first established diagnosis) in individuals with BPD to the year after established depression diagnosis. *Statistically significant difference; ns: no statistical significance; dotted line: time of established diagnosis; CNS: Central nervous system; meds: medications; AHS: anxiolytics, hypnotics, and sedatives; ADHD: Attention deficit/hyperactivity disorder; SSRIs: Selective serotonin reuptake inhibitors; SNRIs: Serotonin and norepinephrine reuptake inhibitors. For numeric presentation, see Supplementary Table 3.1 and 3.2. For presentation of all three patterns of use, see Supplementary Figure 2.

For prevalent use, there was an increase at statistically significant levels (i.e., *p* < 0.001, the FWER adjusted significance level according to Hochberg, see methods section) from the year before to the first year after diagnosis for “any CNS-acting medications” as well as for most of the separate main and subgroups of medication. Exceptions were AHS (*p* = 0.037), antidepressants (*p* = 0.338), opioids (*p* = 0.265), and antiparkinson medication (*p* = 0.006), which were used to a similar extent, and SSRIs, which decreased in use (*p* < 5e-15). From the first year after diagnosis to the third year after diagnosis there was a decrease in use of most groups (all *p* < 0.001), and no statistically significant change for the other groups, which were opioids (*p* = 0.031), ADHD medications (*p* = 0.231), substance use disorder medications (*p* = 0.077), antiparkinson medications (*p* = 0.831), and lithium (*p* = 0.016) (Figure 1 and Supplementary Figure 2 and Supplementary Table 3.1).

##### Duration of use

All medication groups were increasingly long-term used (>12 months), from the year before diagnosis to the year after, with all changes at statistically significant levels except for opioids (*p* = 0.177), and antiparkinson medications (*p* = 0.007). Thereafter, there was a decrease in long-term use from the first year after, to the third year after diagnosis for “any CNS-acting medications,” AHS, antidepressants, and the subgroup SSRI (all *p* < 0.001). Long-term use increased for four groups (i.e., opioids, ADHD medications, first-generation antipsychotics, and lithium, all *p* < 0.001), although increases were very minor. ADHD medication long-term use increased the most from 10.4% the first year after diagnosis to 11.8% the third year after diagnosis (Supplementary Figure 2, Supplementary Table 3.5).

##### Co-medication

The mean level of co-medication increased from the year before to the year after the first established BPD diagnosis, with 34.8% using three or more of the eight main groups of medication and 2.9% using five or more. Co-medication then decreased to the third year after diagnosis. Both changes were statistically significant (*p* < 0.001). The five most used co-medication profiles did not change and included antidepressants and AHS, each alone or combined, and also AHS combined with antidepressants plus antipsychotics or antiepileptics/mood stabilisers. In the year after being diagnosed, there was a peak in individuals using varying combinations (Supplementary Figure 2, Supplementary Table 3.9).

#### Comparison with reference group

##### Prevalence of use

In comparison with the depression group, the BPD group showed more prevalent use of “any CNS-acting medications” and all other groups of medications, except for antidepressants and SSRIs, which were used more in the depression group in the year after diagnosis (all *p* < 0.001; Figure 1, Supplementary Figure 2, and Supplementary Tables 3.1, 3.2). The difference in the prevalence of “any CNS-acting medications” between the BPD and the depression groups was small (90.3% vs 87.5% users), but for the separate medication groups, differences were more pronounced. For instance, differences (BPD to depression in brackets) were more pronounced in AHS (66.0%/53.7%), antipsychotics (36.1%/10.7%), antiepileptics/mood stabilisers (35.5%/6.6%), benzodiazepines (43.1%/26.4%), and lamotrigine (21.4%/4.7%). For medication groups commonly used to treat depression, the difference was also pronounced, that is, antidepressants (70.0%/79.5%) and SSRIs (59.9%/64.9%; Figure 1, Supplementary Figure 2, and Supplementary Tables 3.1 and 3.2).

##### Duration of use

All groups of CNS-acting medications were more frequently used long-term in patients with BPD compared to those with depression, except for SSRIs, which did not significantly differ (*p* = 0.465) (Supplementary Figure 2 and Supplementary Tables 3.5, 3.6).

##### Co-medication

The mean level of co-medication was statistically significantly higher in the BPD group as compared to the depression group (Supplementary Figure 2 and Supplementary Tables 3.9, 3.10).

#### Patterns over follow-up years (2006, 2010, 2014, and 2019) after BPD diagnosis

**Figure 2.**
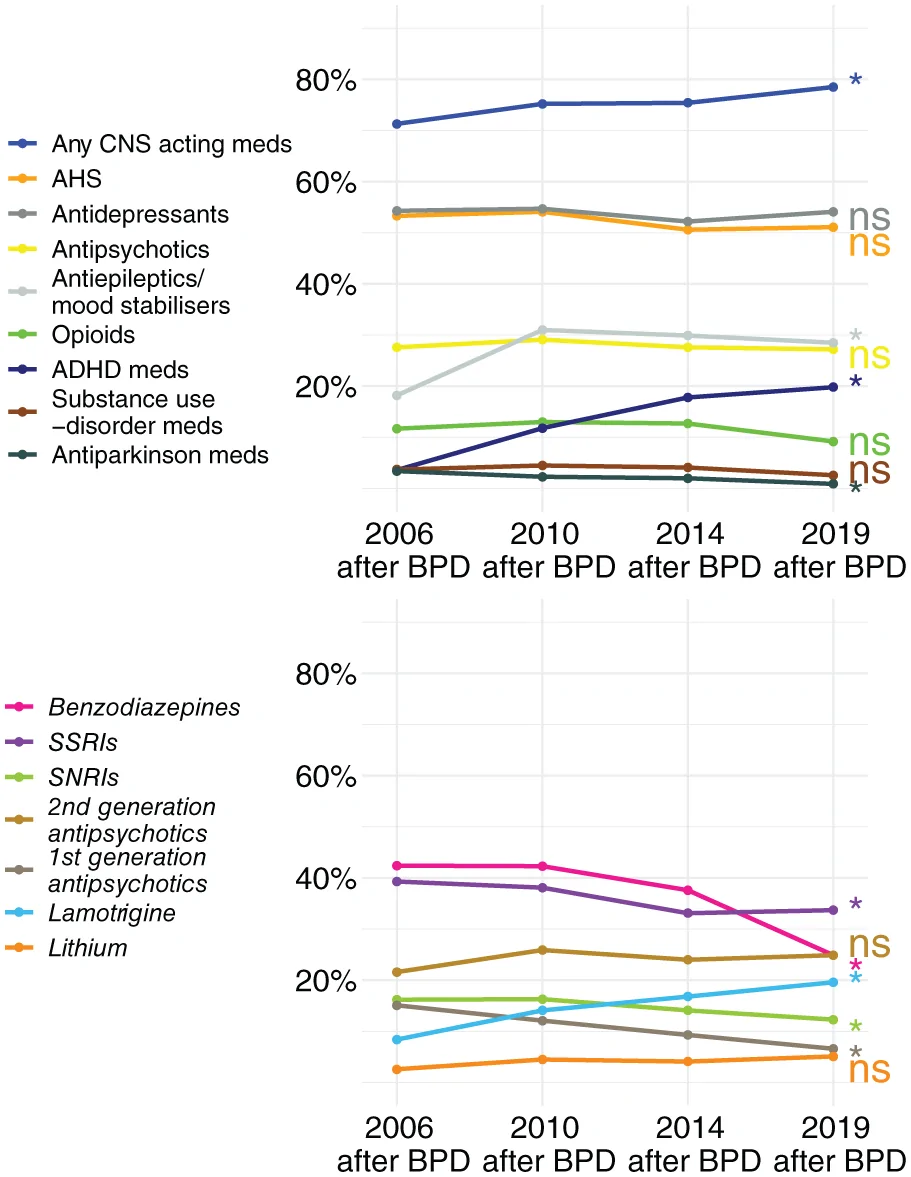
Prevalent medication use patterns in individuals who were young adults in 2006, 2010, 2014, and 2019, and diagnosed with BPD prior to those years. *Note*. Linear regressions of prevalent medication use for change over time. *Statistical significance;ns: no statistical significance; CNS: Central nervous system; meds: medications; AHS: anxiolytics, hypnotics, and sedatives; ADHD: Attention deficit/hyperactivity disorder; SSRIs: Selective serotonin reuptake inhibitors; SNRIs: Serotonin and norepinephrine reuptake inhibitors. For numeric presentation, see Supplementary Table 3.4. For presentation of all three patterns of use, see Supplementary Figure 5.

When assessing changes in the three patterns of use over calendar years (2006, 2010, 2014, and 2019), in individuals who were young adults in those years, an almost monotone increase in prevalent use, long-term use and mean degree of co-medication was found when disregarding the time/age when the BPD diagnosis was established (Supplementary Figure 3 and Supplementary Tables 3.3, 3.7, 3.11). When restricting that group to include only the subgroup of young adults diagnosed with BPD prior to those years (2006, 2010, 2014, and 2019), increases were less pronounced (Figure 2 and Supplementary Figure 4 and Supplementary Tables 3.4, 3.8, 3.12).

##### Prevalence of use

In the restricted, priorly diagnosed, young adult subgroup (i.e., those who had received a first BPD diagnosis at any time before each of those specific sample years) there was an increase in the use of “any CNS-acting medication”; from 71.3% in 2006 to 78.5% in 2019 (*p* = 1e-5, see Figure 2 and Supplementary Figure 4, Supplementary Table 3.4). When examining the different medication groups separately, increases at statistically significant levels (*p* < 0.001) were found for antiepileptics/mood stabilisers, ADHD medications, and lamotrigine. Decreasing use at statistically significant levels was seen for antiparkinson medications, benzodiazepines, SSRIs, SNRIs, and first-generation antipsychotics (all *p* < 0.001). The other groups were each used at similar levels between 2006 and 2019: AHS was used by approximately 55% (*p* = 0.043), antidepressants by 54% (*p* = 0.627), antipsychotics by 27% (*p* = 0.356), whereas second-generation antipsychotics by 26% (*p* = 0.301), opioids by 12% (*p* = 0.001), substance use disorder medications by 5% (0.004), and lithium by 4% (*p* = 0.003; Figure 2 and Supplementary Figure 4, Table 3.4).

##### Duration of use

Long-term use increased for “any CNS-acting medications” over time in the restricted young adult subgroup. Among the separate groups of medication, antiepileptics/mood stabilisers, ADHD medications, and lamotrigine showed increases in long-term use (all *p* < 0.001). In contrast, benzodiazepines and first-generation antipsychotics decreased in long-term use (*p* < 0.001), while other groups showed no statistically significant changes (Supplementary Figure 4 and Supplementary Table 3.8).

##### Co-medication

The mean level of co-medication did not statistically significantly change during the investigated years (2006, 2010, 2014, and 2019). The proportion of individuals using three or more of the main groups of medication for >30 days, however, peaked in 2010 and then decreased slightly. The most common profiles of medication used were AHS and antidepressants alone and in combination (Supplementary Figure 4 and Supplementary Table 3.12).

## Discussion

The present study examined CNS-acting medication use between 2006 and 2019 in a nationwide, population-based cohort of 18,426 young adults diagnosed with BPD, focusing on use in the years around the time of the first established diagnosis. Several important findings emerged with regards to patterns of medication use and changes over time.

First, in young adults, the time when being diagnosed with BPD appears crucial for how medications are used, since prevalent use, long-term use, and co-medication use all peaked during the year following the first registered BPD diagnosis. Almost all young adults in this study (90.3%) used at least one CNS-acting medication in the year following diagnosis. The increase in use following being diagnosed may appear counterintuitive, given the expectation that once a BPD diagnosis is established, adherence to treatment guidelines would increase. This consideration also motivated the choice of the year after the first diagnosis as the reference period for comparisons. Clinical guidelines recommend that pharmacological treatment be reserved for comorbid conditions and in severe crises, that medications with addictive potential be avoided, and that ongoing pharmacotherapy be regularly reviewed and discontinued if no clear benefit is observed. Once a BPD diagnosis is established and BPD-specific psychotherapeutic treatment is initiated, the need for pharmacotherapy would also be expected to be reduced. However, it is also possible that upon commencing evidence-based psychotherapy (e.g., dialectic behaviou therapy or mentalisation-based treatment) and abstaining from medication misuse (such as with non-prescribed and/or illegally obtained), a common prerequisite, patients may experience a transient intensification of negative affect. The initial increase in use could be a strategy to clinically handle emerging emotional dysregulation. It is also possible that frequently scheduled visits and adapted clinical management facilitate an increased, yet safe and beneficial medication use pattern, which could explain the peak in the year after being diagnosed. For instance, our results show that medication for ADHD is used at stable rates and for long durations after being diagnosed with BPD. With ADHD being a common comorbid condition in BPD (Tate et al., 2022), increased use of ADHD medications may improve the ability to maintain focus and to attend sessions, and hence enable benefits from psychotherapy. Moreover, there may be a delay between diagnosis and access to evidence-based psychotherapy due to the limited availability of such treatments, which can contribute to the worsening of symptoms. In this context, medication may be used to manage severe symptoms and acute crises, reflecting both the lack of preferred treatment options for this patient group and pressure on clinicians to provide timely solutions.

Second, comparing medication use in individuals diagnosed with BPD to those diagnosed with depression in psychiatric care helps to contextualise and quantify the extent of medication use. We chose this comparison group because both conditions are frequently associated with psychiatric and non-psychiatric comorbidity, and therefore have similarities in health care needs (Lundberg et al., 2022; Tate et al., 2022). In addition, pharmacological treatment of depression is common (79%) and familiar to many clinicians (Lundberg et al., 2022). Our comparisons indicate high medication use among individuals with BPD. As expected, we found that antidepressants and SSRIs were used more prevalently among individuals with depression in the year after their first registered diagnosis, while all other groups of medications were used more in individuals diagnosed with BPD in the year following being diagnosed.

Finally, the findings indicate that patterns of use have changed over calendar years. Specifically, we observed an overall increase in medication use across calendar years (2006, 2010, 2014, and 2019) among young adults when medication use was examined irrespective of time since, or age at diagnosis. This is important when BPD is conceptualised as a stable, life-long profile that fluctuates over time (“waxing and waning”; Torgersen, 2009; Videler et al., 2019). In clinical practice, referral for evaluation of BPD typically occurs only once symptoms reach a critical threshold. Accordingly, we regarded the restricted subgroup of young adults who had been diagnosed with BPD prior to each index year (2006, 2010, 2014, 2019) as a more homogeneous and informative cohort to investigate for medication use over calendar years (Figure 2 and Supplementary Figure 4, Supplementary Tables 3.4, 3.8, and 3.12). In this group, the overall increase in medication use was more modest, with some medication groups increasingly and others decreasingly used or used to similar extents over calendar years. The most marked change was observed for ADHD medication use, which increased by 5.5-fold from 3.6% to 19.8% users. This pattern parallels the rise in ADHD medication use in the general Swedish population over the same time period (National Board of Health and Welfare, 2023), although from a higher initial level in the BPD group. The use of antiepileptics/mood stabilisers, and particularly lamotrigine, also increased steadily between 2006 and 2019. This is in accordance with a 2010 Cochrane report on the effects of pharmacological treatment in BPD, stating that mood stabilisers and second-generation antipsychotics may be effective for treating core symptoms of BPD and associated psychopathology (Stoffers et al., 2010). However, this pattern contrasts with findings from a randomised controlled trial published in 2018 (Crawford et al., 2018), which found no difference between lamotrigine and placebo in reducing BPD symptoms. In the restricted subgroup of young adults (assessed in 2006, 2010, 2014, and 2019 after first BPD diagnosis), benzodiazepine use—a major class of medications with addictive potential—decreased markedly from 42.4% to 24.9% (*p* = 6e-38). Opioid use, another major group of medications with addictive potential, decreased from 11.7% to 9.2% over the same period, although this change did not reach statistically significant levels (*p* = 0.001). These reductions in benzodiazepine- and opioid use are noteworthy, considering previous reports (Muller et al., 2019) of increasing opioid prescriptions and severe consequences related to opioid and benzodiazepine use (Gibbons et al., 2021). The declining benzodiazepine and opioid use is consistent with Swedish clinical guidelines for personality disorders, which advise against pharmacological agents with addictive potential (Ekselius, 2017). It also mirrors declines in opioid and benzodiazepine use in the total Swedish population during the 2010s, including specifically among young adults (Swedish Medical Agency, 2020, 2021). Another finding of interest was that, in contrast to the previous Spanish study by Pascual et al. 2021 that found higher and increasing rates of second-generation antipsychotic use, we found a stable extent of use (approximately 25%) over time (2006, 2010, 2014, and 2019). This may be due to our study focusing on young adults and not individuals of any age. The initial increase in the Spanish study was hypothesised to be due to broadened indications. When they investigated the later time period, corresponding to a time period similar to the one in our study, the rate of use was stable in the study by Pascual et al., too.

To be noted, a Cochrane report from 2022 (published after the end of the follow-up time of our study) on pharmacological treatments in BPD, concluded that compared to placebo, mood stabilisers are not different in their effect on either BPD symptom severity, self-harm, suicide-related outcomes, or psychosocial functioning (Stoffers-Winterling et al., 2022a). The partly contradictory conclusions between the 2010 and 2022 Cochrane reports (Stoffers et al., 2010; Stoffers-Winterling et al., 2022a) are illustrative of the weak evidence base (few and small studies) on medication effects in BPD.

Taken together, the risks with medication use in BPD, the limited evidence supporting its efficacy, and our findings showing that nearly all young adults with BPD use CNS-acting medication (with the use is increasing over time), underscore the need for clinicians to be familiar with clinical recommendations for BPD, and to adjust care accordingly when considering medication for individuals with BPD. Specifically, the risks associated with medication use, together with the findings from our study, indicate that clinicians should continuously evaluate the risks and benefits of the medications used, especially medications with addictive and misuse potential. They further indicate the importance of facilitating adapted care to individuals with BPD symptomatology (e.g., to schedule follow-up whenever medication is provided, to dispense limited quantities, and to validate suffering in acute crises) to reduce the risks of negative effects, such as iatrogenic addiction and intoxication. Moreover, given the limited evidence base, selecting an appropriate medication remains challenging. Combined with our findings, this highlights the need for easy-to-use and up-to-date guidance for best practice, such as in regularly updated clinical recommendations about BPD.

Future studies should investigate the specific medications within the groups. Furthermore, examining the varying effects and potential side-effects of specific medications, for example, within the anxiolytics, hypnotics, and sedatives, the second-generation antipsychotic, and the ADHD medication groups, would be valuable, as well as adjunct psychotherapeutic treatment to understand their effects on medication use. Further studies of the underlying indications for the prescribed medications are needed to better understand the rationale behind the high and increasing degree of CNS-acting medication use.

The strengths of this study include the national coverage of individuals diagnosed with BPD in specialist care, the long follow-up time from 2006 to mid-2019, the extensive and detailed information on dispensed medications, and the inclusion of a reference group of individuals diagnosed with depression in psychiatric specialist care. Limitations include that our study lacks detailed information on indications for the medications used. For instance, we do not have information on diagnoses established in primary care or information regarding crises. Hence, we lack information on whether prescriptions and use were in accordance with the clinical guidelines (for comorbid conditions diagnosed in primary care and in crises). We also lack information on any psychotherapy received (which would be important to understand its effects on medication use). Another limitation is that we cannot be certain that the dispensed prescriptions have been used, and we do not have information on medication provided by other means (such as during hospital admissions), although we consider our data of filled prescriptions to be indicative of patterns of use.

## Conclusions

The results of the current study suggest that in the young adult group (aged 18–24) with BPD in Sweden, the time of diagnosis was crucial for how medications were used. Medication use peaked during the year following the first registered BPD diagnosis for prevalent use (90.3%), for long-term use (74.5%), and for co-medication (34.8% used ⩾3 groups in co-medication). CNS-acting medication use was more extensive in individuals with BPD compared to individuals with depression in the year following the first diagnosis, and the extent of medication use increased over calendar years (2006–2019). Our results point to the importance of continuously evaluating risks and benefits with medication use in young adults diagnosed with BPD within the clinical context to facilitate appropriate, balanced, and safe medication use in this vulnerable patient population. It also highlights the importance of accumulating more knowledge on risks and benefits with medication use in individuals with BPD, and that the knowledge is continuously made easily accessible to clinicians, for instance, in clinical guidelines, to be of greatest benefit.

## Supplemental Material

sj-docx-1-jop-10.1177_02698811251413479 – Supplemental material for Central nervous system acting medication used by young adults with borderline personality disorder in Sweden 2006–2019 Supplemental material, sj-docx-1-jop-10.1177_02698811251413479 for Central nervous system acting medication used by young adults with borderline personality disorder in Sweden 2006–2019 by Annika Tiger, Yasmina Molero, Isabell Brikell, Zheng Chang, Paul Lichtenstein, Henrik Larsson, Brian M D’Onofrio, Clara Hellner, Tyra Lagerberg, Nitya Jayaram-Lindström and Ralf Kuja-Halkola in Journal of Psychopharmacology

sj-xlsx-2-jop-10.1177_02698811251413479 – Supplemental material for Central nervous system acting medication used by young adults with borderline personality disorder in Sweden 2006–2019 Supplemental material, sj-xlsx-2-jop-10.1177_02698811251413479 for Central nervous system acting medication used by young adults with borderline personality disorder in Sweden 2006–2019 by Annika Tiger, Yasmina Molero, Isabell Brikell, Zheng Chang, Paul Lichtenstein, Henrik Larsson, Brian M D’Onofrio, Clara Hellner, Tyra Lagerberg, Nitya Jayaram-Lindström and Ralf Kuja-Halkola in Journal of Psychopharmacology
