# Supplementary material for: Central nervous system acting medication used by young adults with borderline personality disorder in Sweden 2006–2019
Source: J Psychopharmacol. 2026 Feb 16;40(9):1343–51. doi: 10.1177/02698811251413479 (PMC13490660; doi:10.1177/02698811251413479)
Supplement: Supplementary file 1 [file sj-docx-1-jop-10.1177_02698811251413479.docx]

**Central nervous system acting medication used by young adults with borderline personality disorder in Sweden 2006-2019**

**Journal:** Journal of Psychopharmacology

**Authors:** Annika Tiger, Yasmina Molero, Isabell Brikell, Zheng Chang, Paul Lichtenstein, Henrik Larsson, Brian M D’Onofrio, Clara Hellner, Tyra Lagerberg, Nitya Jayaram- Lindström, Ralf Kuja-Halkola

**Corresponding author contact details**: Annika Tiger, Centre for Psychiatry Research, Department of Clinical Neuroscience, Karolinska Institutet & Stockholm Health Care Services, Region Stockholm, Norra Stationsgatan 69, 113 64 Stockholm. E-mail: [annika.tiger@ki.se](mailto:annika.tiger@ki.se)

**Supplementary Figure 1.** Cohort selection

**Supplementary Table 1.** Medication groups and ATC-codes included in each group, with NbN-terminology indicated where applicable (in blue)

Main groups:

|  |  | **NbN** | |  |
| --- | --- | --- | --- | --- |
| **Medication group** | **ATC-codes** | **Pharmacology** | **Mode of action** | **Drugs** |
| **Anxiolytics, hypnotics and sedatives** | N05B or N05C | Drugs for anxiety and insomnia | NA | all drugs within indicated ATC-codes |
|  | N05BA01, N05BA02,  N05BA04, N05BA06,  N05BA08, N05BA11,  N05BA12, N05BA19,  N05BA21, N05CD01, N05CD02, N05CD03, N05CD04, N05CD05, N05CD06, N05CD07, N05CD08, N05CD10, N05CF01, N05CF02,  N05CF03, N05CF04 | GABA | positive allosteric modulator  (GABA-A receptor, benzodiazepine  site) | diazepam, chlordiazepoxide, oxazepam, lorazepam, broazepam, prazepam,  alprazolam, etizolam,  clotizaepam, flurazepam,  nitrazepam, flunitrazepam,  estazolam, triazolam,  lormetazepam, temazepam,  midazolam, quazepam,  zopiclone, zolpidem,  zaleplon, eszopiclone |
|  | N05BB01 | histamine | receptor antagonist (H1) | hydroxizine |
|  | N05BE01 | serotonin | receptor partial agonist (5-HT1A) | buspirone |
|  | N05CH01, N05CH02 | melatonin | receptor agonist (M1, M2) | melatonin, ramelteon |
|  | N05CJ01 | orexin | receptor antagonist | suvorexant, lemborexant |
|  | N05CM02 | GABA | positive allosteric modulator (GABA-A receptor, barbiturate site) | clomethiazole |
| **Antidepressants** | N06A | Drugs for depression | NA | all drugs within indicated ATC-codes |
|  | N06AA01, N06AA10,  N06AA21, N06AC01,  N06AX18, | norepinephrine | reuptake inhibitor (NET) | desipramine, nortryptyline,  maprotiline,  reboxetine |
|  | N06AA02, N06AA04,  N06AA07, N06AA16,  N06AA17, N06AE06,  N06AX16, N06AX17,  N06AX21, N06AX23,  N06AX28 | serotonin, norepinephrine | reuptake inhibitor (SERT NET) | imipramine, clomipramine,  lofepramine, dosulepin,  amoxapine, venlafaxine,  venlafaxine, milnacipran,  duloxetine, desvenlafaxine,  levomilnacipran |
|  | N06AA06 | serotonin, dopamine | receptor antagonist (5-HT2 D2) | trimipramine |
|  | N06AA09 | serotonin, norepinephrine | reuptake inhibitor, receptor antagonist (5-HT2) | amitriptyline |
|  | N06AA12 | serotonin, norepinephrine | reuptake inhibitor (NET, SERT), receptor antagonist (5-HT2) | doxepin |
|  | N06AB03, N06AB04,  N06AB05, N06AB08,  N06AB10, N06AE02 | serotonin | reuptake inhibitor (SERT) | fluoxetine, citalopram,  paroxetine, sertraline,  escitalopram, fluvoxamine |
|  | N06AC03, N06AX11 | norepinephrine, serotonin | receptor antagonist (NE alpha-2, 5HT2, 5-HT3) | mirtazapine |
|  | N06AC02, N06AX03 | norepinephrine | receptor antagonist (alphra-2), reuptake inhibitor (NET) | mianserin |
|  | N06AF01, N06AF03 | serotonin, norepinephrine, dopamine | enzyme inhibitor (MAO-A, and -B) | isocarboxazid, phenelzine |
|  | N06AF04 | serotonin, norepinephrine, dopamine | enzyme inhibitor (MAO-A, and -B), release (DA, NE) | tranycypromine |
|  | N06AG02 | serotonin, norepinephrine, dopamine | reversible enzyme inhibitor (MAO-A) | moclobemide |
|  | N06AX05 | serotonin | serotonin reuptake inhibitor (SERT), receptor agonist (5-HT1A), receptor antagonist (5HT2) | trazodone |
|  | N06AX06 | serotonin | receptor antagonist (5-HT2), receptor agonist (5-HT1A) | nefazodone |
|  | N06AX12 | norepinephrine, dopamine | reuptake inhibitor (NET, DAT), releaser (NE, DA) | bupropion |
|  | N06AX14 | glutamate, opioid | unclear mode of action | tianpentine |
|  | N06AX22 | melatonin, serotonin | receptor agonist (Mel1, Mel2), receptor antagonist (5-HT2B, 5-HT2C) | agomelatine |
|  | N06AX24, | serotonin | reuptake inhibitor (SERT), receptor partial agonist (5-HT1A) | vilazodone |
|  | N06AX26, | serotonin | reuptake inhibitor (SERT), receptor partial agonist (5-HT1A), receptor antagonist (5-HT3) | vortioxetine |
|  | N06AX27 | glutamate | NMDA antagonist | esketamine |
|  | N06AX29 | GABA | PAM (GABA-A receptor, neurosteroid site) | brexanolone |
| **Antipsychotics** | N05A, except N05AN01 (=Lithium) | Drugs for psychosis | NA | all drugs within indicated ATC-codes |
|  | N05AA02, N05AC02,  N05AC04, N05AF01,  N05AF03, N05AH01,  N05AE03, N05AE04,  N05AE05, N05AH03,  N05AX11, N05AX14 | dopamine, serotonin | receptor antagonist (D2, 5HT2) | levomepromazine, thioridazine, popotiazine, flupenthixol, chlorprothixene,  loxapine, sertindole, ziprasidone, lurasidone,  olanzapine, zotepine,  iloperidone |
|  | N05AB3, N05AC01,  N05AD01, N05AF05,  N05AG02, N05AL03,  N05AL01, N05AL05 | dopamine | receptor antagonist (D2) | perphenazine, periciazine,  haloperidol, zuclopenthixol,  pimozide, tiapride,  sulpiride, amisulpiride |
|  | N05AH02, N05AX08,  N05AX13 | dopamine, serotonin, noradrenaline | receptor antagonist (D2, 5-HT2, NE  alpha-2) | clozapine, risperidone,  paliperidone |
|  | N05AH04 | dopamine, serotonin, noradrenaline | receptor antagonist (D2,  5-HT2) and reuptake inhibitor (NET)  (metabolite) | quetiapine |
| **Antiepileptics and mood stabilisers** | N03A and N05AN01 (=Lithium) | Drugs for relapse prevention | NA | all drugs within indicated ATC-codes |
|  | N03AE01 | GABA | positive allosteric modulator (GABA-A receptor, benzodiazepine site) | clonazepam |
|  | N03AF01, N03AF02 | flutamate | voltage-gated sodium and calcium channel blocker | carbamazepine, oxcarbamazepine |
|  | N03AG01 | glutamate | unclear | valproate |
|  | N03AX09 | glutamate | voltage-gated sodium channel blocker | lamotrigine |
|  | N03AX11 | GABA, glutamate | facilitation of GABA transmission, receptor antagonist on AMPA and KA | topiramate |
|  | N05AN01 | Lithium | enzyme modulator | lithium |
| **Opioids ^a^** | N02A | NA | NA | all drugs within indicated ATC-codes |
|  | N02AE01 | opioid | receptor partial agonist (mu), receptor antagonist (kappa, delta) | buprenorphine |
| **ADHD medications** | N06B and C02AC02 | NA | NA | all drugs within indicated ATC-codes |
|  | N06BA01, N06BA02,  N06BA04, N06BA12, | norepinephrine, dopamine | reuptake inhibitors (DAT, NET), release (DA, NE) | amphetamine, methylphenidate, lisdexamphetamine |
|  | N06BA07, | dopamine | reuptake inhibitor (DAT) | modafinil |
|  | N06BA09, | norepinephrine | reuptake inhibitor (NET) | atomoxetine |
|  | C02AC02 | norepinephrine | receptor agonist (alpha-2 NE) | guanfacine |
| **Substance related disorder medications (includes substances that can be used to treat severe pain)** | N07B | NA | NA | all drugs within indicated ATC-codes |
|  | N07BA02 | norepinephrine, dopamine | reuptake inhibitors (DAT, NET), release (DA, NE) | bupropion |
|  | N07BA03 | acetylcholine | receptor partial agonist (nicotinic receptors) | varenicline |
|  | N07BB01 | alcohol | enzyme inhibitor | disulfiram |
|  | N07BB03 | glutamate | unclear | acamprosate |
|  | N07BB04 | opioid | receptor antagonist (mu, kappa) | naltrexone |
|  | N07BB05 | opioid | receptor antagonist (mu,delta) receptor partial agonist (kappa) | nalmefen |
|  | N07BC01 | opioid | receptor partial agonist (mu), receptor antagonist (kappa,delta) | buprenorphine |
|  | N07BC02 | opioid | receptor agonist (mu) | methadone |
|  | N07BC04 | norepinephrine | receptor agonist (alpha-2 NE) | lofexidine |
| **Antiparkinson medications ^b^** | N04 | NA | NA | all drugs within indicated ATC-codes |
|  | N04BC05 | dopamine | receptor agonist (D2) | pramipexol |
|  | N04BD01 | dopamine, norepinephrine, serotonin | enzyme inhibitor (MAO-B and -A) | selegiline |
| **Any CNS acting medications** | Any of the above | NA | NA | Any of the above |

Subgroups, medications of specific interest:

|  |  | **NbN** | |  |
| --- | --- | --- | --- | --- |
| **Medication group** | **ATC-codes** | **Pharmacology** | **Mode of action** | **Drugs** |
| ***Benzodiazepine type medications* ^a^** | N03AE, N05BA, N05CD, N05CF | NA | NA | all drugs within indicated ATC-codes |
|  | N03AE01, N05BA01, N05BA02, N05BA04, N05BA06, N05BA08, N05BA11, N05BA12, N05BA19, N05BA21, N05CD01, N05CD02, N05CD03, N05CD04, N05CD05, N05CD06, N05CD07, N05CD08, N05CD10, N05CF01, N05CF02, N05CF03, N05CF04 | GABA | positive allosteric modulator  (GABA-A receptor, benzodiazepine  site) | clonazepam, diazepam, chlordiazepoxide, oxazepam, lorazepam, broazepam, prazepam,  alprazolam, etizolam,  clotizaepam, flurazepam,  nitrazepam, flunitrazepam,  estazolam, triazolam,  lormetazepam, temazepam,  midazolam, quazepam,  zopiclone, zolpidem,  zaleplon, eszopiclone |
| ***SSRIs ^c^*** | N06AB | NA | NA | all drugs within indicated ATC-codes |
|  | N06AB03, N06AB04,  N06AB05, N06AB06,  N06AB08, N06AB10 | serotonin | reuptake inhibitor (SERT) | fluoxetine, citalopram, paroxetine, sertraline, fluvoxamine, escitalopram, |
| ***SNRIs ^c^*** | N06AX16, N06AX17, N06AX21 | NA | NA | all drugs within indicated ATC-codes |
|  | N06AX16, N06AX17, N06AX21 | serotonin, norepinephrine | reuptake inhibitor (SERT and NET) | venlafaxine, milnacipran,  duloxetine |
| ***Second generation antipsychotics ^d^*** | N05AE03-N05AE05, N05AH02-N05AH05, N05AL01-N05AL02, N05AL04-N05AL05, N05AL07, N05AX08, N05AX10-N05AX16 | NA | NA | all drugs within indicated ATC-codes |
|  | N05AE03, N05AE04,  N05AE05, N05AH03,  N05AX11, N05AX14, | dopamine, serotonin | receptor antagonist (D2, 5HT2) | sertindole, ziprasidon, lurasidone, olanzapine,  zotepine, iloperidon |
|  | N05AX12, N05AX16 | dopamine, serotonin | receptor partial agonist (D2, 5-HT2) and receptor antagonist (5-HT2) | aripiprazole, brexpiprazol |
|  | N05AH02, N05AX08,  N05AX13 | dopamine, serotonin, noradrenaline | receptor antagonist (D2, 5-HT2, NE  alpha-2) | clozapine, risperidone,  paliperidone |
|  | N05AH04 | dopamine, serotonin, noradrenaline | receptor antagonist (D2,  5-HT2) and reuptake inhibitor (NET)  (metabolite) | quetiapine |
|  | N05AL01, N05AL05 | dopamine | receptor antagonist (D2) | sulpiride, amisulpiride |
| ***First generation antipsychotics ^d^*** | N05AA01-N05AA07, N05AB01-N05AB10, N05AC01-N05AC04, N05AD01-N05AD09, N05AE01-N05AE02, N05AF01-N05AF05, N05AG01-N05AG03, N05AH01, N05AH06, N05AL03, N05AL06, N05AX07 | NA | NA | all drugs within indicated ATC-codes |
|  | N05AA02, N05AB06 N05AC02,  N05AC04, N05AF01,  N05AF03, N05AH01, | dopamine, serotonin | receptor antagonist (D2, 5HT2) | levomepromazine, trifluoperazine, thioridazine, pipotiazine, flupenthixol, chlorprothixene, loxapine |
|  | N05AB3, N05AC01,  N05AD01, N05AF05,  N05AG02, N05AL03 | dopamine | receptor antagonist (D2) | perphenazine, periciazine, haloperidol, zuclopenthixol, pimozide, tiapride |
| ***Lamotrigine ^e^*** | N03AX09 | glutamate | voltage-gated sodium channel blocker | lamotrigine |
| ***Lithium ^f^*** | N05AN01 | lithium | enzyme modulator | lithium |

Note: **ADHD**=Attention deficit-/hyperactivity disorder, **CNS**=central nervous system, **SSRIs**=Selective serotonin reuptake inhibitors, **SNRIs**=Serotonin and norepinephrine reuptake inhibitors.

*^a^ =major groups of CNS acting medications with addictive potential, ^b^=* Includes substances used to treat side effects from antipsychotics*, ^c^=major subgroups of antidepressants, ^d^=major subgroups of antipsychotics, ^e^=* previously considered effective and safe in alleviating symptoms of BPD but an RCT from 2018 [1] indicating it not better than placebo, *^f^*= common mood stabilizer used in bipolar disorder.

[1]	M. J. Crawford *et al.*, "Lamotrigine for people with borderline personality disorder: a RCT," *Health Technol Assess,* vol. 22, no. 17, pp. 1-68, Apr 2018, doi: 10.3310/hta22170.

**Supplementary Table 2.** Comorbid psychiatric conditions in young adults diagnosed with BPD.

| Investigated population (N) |  | 10,095 | 9,490 |
| --- | --- | --- | --- |
| Comorbid condition | ICD-10 codes | Prevalence in young adults during year before BPD, n (%) | Prevalence in young adults during year after BPD, n (%) |
| ADHD | F90 | 1475 (14.6) | 1984 (20.9) |
| Affective disorders | F34, F38, F39 | 287 (2.8) | 266 (2.8) |
| Anxiety disorders | F40, F41 | 4741 (47) | 3870 (40.8) |
| Autism | F84.0, F84.1, F84.5, F84.8, F84.9 | 403 (4) | 506 (5.3) |
| Bipolar disorders | F30, F31 | 877 (8.7) | 1109 (11.7) |
| Depression | F32, F33 | 3434 (34) | 2718 (28.6) |
| Eating disorders | F50.0, F50.1, F50.3, F50.9 | 753 (7.5) | 862 (9.1) |
| Intellectual disability | F70-F79 | 89 (0.9) | 111 (1.2) |
| OCD | F42 | 321 (3.2) | 345 (3.6) |
| Personality disorder other than BPD | F60 excluding F603 | 1093 (10.8) | 1083 (11.4) |
| PTSD | F431 | 284 (2.8) | 216 (2.3) |
| Psychotic disorders | F20, F21, F25 | 74 (0.7) | 90 (0.9) |
| Substance use disorders | F10- F16, F18-F19 | 1848 (18.3) | 1982 (20.9) |
| Tic disorder | F95.0, F95.1, F95.2, F95.8, F95.9 | 19 (0.2) | 19 (0.2) |
| Any of the above |  | 7692 (76.2) | 7188 (75.7) |

Note: **ADHD**=Attention deficit-/hyperactivity disorder, **OCD**=Obsessive-compulsive disorder, **PTSD**= Posttraumatic stress disorder.

See separate excel file for **Supplementary Table 3.**

**Supplementary Figure 2.** Medication use patterns in individuals diagnosed with BPD, who were young adults in indicated years (i.e., the year before, and 1, 2, and 3 years after first registered BPD diagnosis). Including data for the same years around first depression diagnosis in matched individuals diagnosed with depression in specialist care for comparison.

Note:	**a1/a2:** *Prevalent* use of medication in young adults diagnosed with BPD, **b1/ b2:** *Duration* of use of medication in young adults diagnosed with BPD, **c:** *Co-medication* in young adults diagnosed with BPD

Linear regressions, comparing the reference year (year after first established BPD diagnosis) to the year before and the third year after established BPD diagnosis.

**d1/d2:** *Prevalent* use of medication in young adults diagnosed with *depression*, **e1/ e2:** *Duration* of use of medication in young adults diagnosed with *depression*, **f:** *Co-medication* in young adults diagnosed with *depression*

Linear regression comparing the reference year (the year after first established diagnosis) in young adults with BPD to the same year in the reference group, i.e.: the year after established depression diagnosis.

“*“= statistically significant difference, “ns”= no statistical significance, dotted line = time of established diagnosis, **CNS**=Central nervous system, **meds**=medications, **AHS**=anxiolytics, hypnotics and sedatives, **ADHD**=Attention deficit/hyperactivity disorder, **SSRIs**= Selective serotonin reuptake inhibitors, **SNRIs**=Serotonin and norepinephrine reuptake inhibitors.

For numeric presentation see **Supplementary Table** **3.1, 3.2, 3.5, 3.6, 3.9**, and **3.10**

**Supplementary Figure 3.** Medication use patterns in individuals who were young adults in 2006, 2010, 2014 and 2019, and diagnosed with BPD any time

Note:	**a1/a2**: Prevalent use medication, **b1/b2**: Durations of use of medication, **c**: Co-medication

Linear regression for change over calendar years.

“*****“= statistical significance, “**ns**”= no statistical significance, **CNS**=Central nervous system, **meds**=medications, **AHS**=anxiolytics, hypnotics and sedatives, **ADHD**=Attention deficit/hyperactivity disorder, **SSRIs**=Selective serotonin reuptake inhibitors, **SNRIs**=Serotonin and norepinephrine reuptake inhibitors.

For numeric presentation see **Supplementary Table 3.3, 3.7**, and **3.11.**

**Supplementary Figure 4.** Medication use patterns in individuals who were young adults in 2006, 2010, 2014 and 2019, and diagnosed with BPD prior to those years.

Note:	**a1/a2**: Prevalent use of medication, **b1/b2**: Durations of use of medication, **c**: Co-medication

Linear regression of medication for change over time.

“*****“= statistical significance, “**ns**”= no statistical significance, **CNS**=Central nervous system, **meds**=medications, **AHS**=anxiolytics, hypnotics and sedatives, **ADHD**=Attention deficit/hyperactivity disorder, **SSRIs**=Selective serotonin reuptake inhibitors, **SNRIs**=Serotonin and norepinephrine reuptake inhibitors.

For numeric presentation see **Supplementary Table 3.4, 3.8** and **3.12**.
